# Frequent hospital admission of older people with chronic disease: a cross-sectional survey with telephone follow-up and data linkage

**DOI:** 10.1186/1472-6963-12-373

**Published:** 2012-10-30

**Authors:** Jo M Longman, Margaret I Rolfe, Megan D Passey, Kathy E Heathcote, Dan P Ewald, Therese Dunn, Lesley M Barclay, Geoffrey G Morgan

**Affiliations:** 1 University Centre for Rural Health – University of Sydney, PO Box 3074, Lismore, NSW 2480, Australia; 2 Southern Cross Univeristy, PO Box 157, Lismore, NSW 2480, Australia; 3 North Coast NSW Medicare Local, PO Box 519, Lismore, NSW 2480, Australia; 4 North Coast Area Health Service, Locked Mail Bag 11, Lismore, NSW 2480, Australia; 5 School of Medicine, Griffith University, High St, Southport, Qld 4215, Australia

**Keywords:** Frequent hospitalisation, Frequent admission, Avoidable admission, Potentially preventable admission, Older people, Chronic disease, Rural, Australia, Social isolation.

## Abstract

**Background:**

The continued increase in hospital admissions is a significant and complex issue facing health services. There is little research exploring patient perspectives or examining individual admissions among patients with frequent admissions for chronic ambulatory care sensitive (ACS) conditions. This paper aims to describe characteristics of older, rural patients frequently admitted with ACS conditions and identify factors associated with their admissions from the patient perspective.

**Methods:**

Patients aged 65+ resident in North Coast NSW with three or more admissions for selected ACS chronic conditions within a 12 month period, were invited to participate in a postal survey and follow up telephone call. Survey and telephone data were linked to admission and health service program data. Descriptive statistics were generated for survey respondents; logistic regression models developed to compare characteristics of patients with 3 or with 4+ admissions; and comparisons made between survey respondents and non-respondents.

**Results:**

Survey respondents (n=102) had a mean age of 77.1 years (range 66–95 years), and a mean of 4.1 admissions within 12 months; 49% had at least three chronic conditions; the majority had low socioeconomic status; one in five (22%) reported some difficulty affording their medication; and 35% lived alone. The majority reported psychological distress with 31% having moderate or severe psychological distress. While all had a GP, only 38% reported having a written GP care plan. 22% of those who needed regular help with daily tasks did not have a close friend or relative who regularly cared for them. Factors independently associated with more frequent (n=4+) relative to less frequent (n=3) admissions included having congestive heart failure (p=0.003), higher social isolation scores (p=0.040) and higher Charlson Comorbidity Index scores (p=0.049). Most respondents (61%) felt there was nothing that could have avoided their most recent admission, although some potential avoidability of admission was described around medication and health behaviours. Respondents were younger and less sick than non-respondents.

**Conclusions:**

This study provides a detailed description of older patients with multiple chronic conditions and a history of frequent admission in rural Australia. Our results suggest that programs targeting medication use, health behaviours and social isolation may help reduce multiple hospital admissions for chronic disease.

## Background

The continued increase in hospital admission is a significant and complex issue facing health services in Australia and internationally [1,2]. Frequent hospital admissions that are potentially avoidable stretch health services and budgets as well as patients and their carers/family [3]. Research exploring characteristics of patients with a history of frequent admission has identified healthcare utilisation patterns, individual characteristics and environmental factors as risk factors [2,4-7].

Prior hospital admission is a predictor of readmission and frequent admission [2,8,9]. Frequent admission is associated with older age, being male, having high levels of comorbidity and disease severity, depression, anxiety or psychosis [6,7,10,11]. Environmentally, lack of social support [6,10] and socio-economic deprivation [10,12,13] have been shown to impact on admission patterns, but little is known about how these factors influence admission patterns in rural areas.

Ambulatory Care Sensitive (ACS) chronic conditions are conditions such as diabetes, congestive heart failure (CHF), and chronic obstructive pulmonary disease (COPD) which respond well to care in primary healthcare settings and for which hospital admission may be avoided with quality primary care [14]. Frequent hospital admissions are associated with chronic ACS conditions [2]. Older patients with chronic conditions make the greatest contribution to ACS admissions [14-17].

Hospital admission for ACS conditions is particularly burdensome in rural and remote Australia [14,18-20]. In the North Coast of NSW the rate of admission for ACS conditions from 2001–6 was higher than the NSW rate [21]. The 2006–7 rate of admission for ACS chronic conditions (excluding diabetes) in very remote regions of New South Wales (NSW) was almost double that in major NSW cities [22]. Previous research on ACS admissions has primarily explored urban populations, with little attention to potential differences for rural patients [2,4,6].

Australian research on avoidable admission has generally used ACS conditions as a proxy for avoidability, and has relied on analysis of routinely available hospital admissions data. Classifying a hospital admission for an ACS condition as ‘potentially avoidable’ does not imply that the individual admission *is* actually avoidable. Rather, it is a population measure based on specific admission diagnoses [23]. Further, the timescale of any notion of “avoidability” inherent in an ACS admission is unclear, i.e. was the admission avoidable with primary healthcare intervention on the day/month/year or decades before the admission? Therefore the use of ACS admissions as a proxy for avoidable admissions for planning interventions focused on individuals, presents difficulties. The proportion of ACS admissions that might actually be avoidable remains unknown and little research has explored the avoidability of specific admissions or the antecedents to admission from the perspective of patients [17,24,25].

The NSW Health Connecting Care Program launched in 2010 aimed to improve chronic disease management amongst patients with frequent hospital admissions for selected chronic conditions by improving coordination of their care. Patients eligible for inclusion in the North Coast Connecting Care Program were older patients resident in rural North Coast NSW with 3 or more hospital admissions for ACS chronic condition within a 12 month period.

Our study addressed gaps in current knowledge by investigating this rural patient group, and aiming to: report findings of a cross-sectional survey of this group of patients (describing their demographic, health, management of their condition and social characteristics, and exploring the avoidability of individual admissions, from the patient perspective); and determining factors associated with patients who are frequently admitted (3 admissions in 12 months) compared with those who are very frequently admitted (4+ admissions in 12 months). In addition, we explored whether survey respondents were representative of patients targeted by the Connecting Care Program, by linking patient survey data to hospitalisation and Connecting Care Program data to compare characteristics of the patient group as a whole with the subset of patients who participated in the survey. The results of our study will inform the design of interventions to better manage patients with frequent admissions for chronic disease and potentially reduce hospital admissions within this patient group. Our study obtained ethical approval from the North Coast Area Health Service Human Research Ethics Committee.

## Methods

Our survey targeted patients eligible for inclusion in the North Coast Connecting Care Program, i.e. residents of North Coast NSW with three or more unplanned hospital admissions for selected ACS chronic conditions in any 12 month period from July 2008 to December 2009. The full eligibility criteria are summarised in Table 1. The North Coast of NSW is a 500 kilometre-long coastal strip along the NSW north coast, and shares a state border with Queensland. The population is around half a million, and is mostly concentrated in regional cities and towns with the remaining population scattered across the rural hinterland.

**Table 1:** Eligibility criteria for the North Coast connecting care program

	
*Inclusion characteristics*	Usual residential address within North Coast Area Health Service, and
	Aged over 65 at admission, and
	One or more diagnoses of:
	· Chronic Obstructive Pulmonary Disease (COPD) (ICD10: J41 to J44)
	· Diabetes (ICD10: E10 to E15)
	· Hypertension (ICD10: I10, I11.9)
	· Congestive heart failure (CHF) (ICD10: I50, I11.0, J81)
	· Coronary artery disease (ICD10: I20 to I25)
	At least one unplanned acute admission in any 12 month period between **July 2008 and Dec 2009** with one of the above five diseases as the principal diagnosis and a total of 3 or more unplanned acute hospital admissions in the same 12 month period with one of the five diseases as either the principal diagnosis or an additional diagnosis
*Exclusion characteristics*	In receipt of palliative care
	In a residential aged care facility

Patients were identified as being eligible for the North Coast Connecting Care Program by NSW Health in the first quarter of 2010. Patients were sourced from routinely collected admissions data from North Coast NSW hospitals in the public sector. In October 2010 a letter (including an information sheet and consent form) inviting patients to participate in the research was mailed to all eligible patients, excluding those who were known to have died (101 patients) or moved to an aged care facility (96 patients). Patients who did not respond received a second invitation 2–3 weeks later. Patients who granted consent were mailed the survey questionnaire (and a stamped addressed return envelope) and asked to return the completed survey by post.

**Figure 1:**
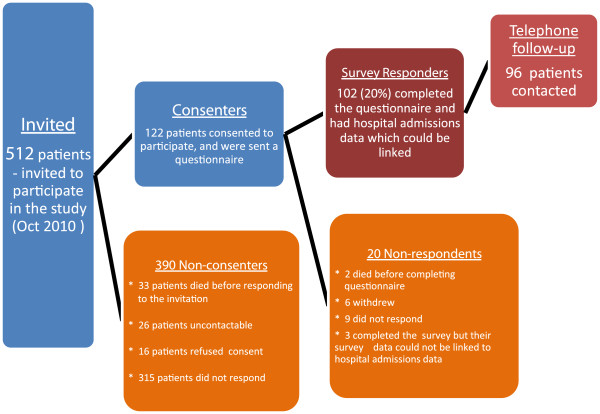
Recruitment for the Survey.

Patients who consented to participate in the survey but did not return a completed questionnaire were mailed the survey again. Consenting patients who still had not responded were telephoned to ask for return of the completed questionnaire, and a number of participants were sent the questionnaire again at this point. Figure 1 summarises recruitment for the survey.

### Questionnaire

The questionnaire was informed by a number of sources: the literature on frequent and avoidable admission, our earlier scoping study of a group of key informants from community based services working with this patient group [26], and advice from the project steering committee that included clinicians, health service managers and policy makers. The questionnaire was also designed to complement the hospitalisation data collected for these patients.

We used validated survey questions where possible. Many of these items were from standard questions in Australian Bureau of Statistics (ABS) Health Surveys and from large Australian cohort studies e.g. the 45 and Up Study [27], and the Australian Longitudinal Study on Women's Health [28].

The questionnaire covered: functional health and well-being; social network (the abbreviated form of the Duke Social Support Index using four items on the size of respondents’ social networks and amount of social contact, with scoring from 4 [lowest network score]-12 [highest]), psychological distress, medication use, whether they had a GP, care plans, disease self-management, carers and care requirements, mobility and access, falls, and demographic characteristics. A more detailed description of these measures can be found in Additional file 1: Appendix 1.

### Follow-up telephone calls

We attempted to contact all survey respondents for a follow-up phone call. This call lasted approximately 10 minutes, and addressed any gaps in the questionnaire answers, checked any anomalies, and clarified questionnaire responses.

In addition, the following open ended questions about patients’ most recent unplanned admission to hospital were asked:

What made you decide that you needed help?

What could have helped you at that time?

Would it have kept you out of hospital?

### Data linkage and analysis

Hospital admissions data for patients in the group invited to participate in the survey (n=512) were obtained from NSW Health for an eighteen month period from July 2008 to December 2009. We selected only unplanned emergency hospital admissions within 12 months of a patient’s index admission. The index admission was defined as the first admission within any 12 month period from July 2008 to December 2009 in which the patient satisfied the inclusion characteristics specified in Table 1.

The Charlson Comorbidity Index (CCI) was calculated for each selected admission [29-31]. The CCI is an index of the risk of mortality from comorbidity during the next 12 months and calculates a score from secondary diagnoses of admissions weighted for type of condition. The maximum CCI score, over all admissions in the index year for each individual patient was used for analysis, and scores were categorised in a standard way into three groups (0, 1–2, 3 or more) [30,32,33].

Multi-morbidity is a score of the number of chronic diseases from the targeted conditions (see Table 1), identified from either the primary or secondary diagnoses for each hospital admission [34,35]. We determined the maximum multi-morbidity score over all admissions in the index year for each individual patient.

The North Coast Connecting Care Program collected data on patients eligible for inclusion in the Program in mid-2010. These data included information on: whether patients had died or moved into residential aged care facilities; GP contact details, and whether the patient was prescribed more than five medications (polypharmacy).

We linked the hospital admissions data and the Connecting Care Program data to the survey data for all patients where this link was possible. This enabled us to compare a range of characteristics of patients in our survey group with those who did not participate in the survey, and to generate a more comprehensive picture than had previously been available.

IBM SPSS Statistics version 19 was used for the majority of the statistical data analysis, with SAS Version 9.3 used to compute the Charlson Comorbidity Index (CCI). Descriptive statistics, Chi-square and odds ratio analyses were used for comparisons between categorical variables. T-tests and analysis of variance or general linear models were used for internal comparisons of continuous survey-based variables with gender and age groupings.

The number of admissions per patient can be viewed as count data which although skewed often follows a Poisson distribution. Therefore Poisson regression models with log link were used to directly model comparisons for numbers of admissions [36] rather than using transformed scores. The log link function ensures non-negative outcomes.

Mean length of stay (bed days) comparisons were analysed using generalised estimating equation (GEE) models which allowed for clustering of repeated hospital admissions among patients. The length of stay duration for each patient is likely to be correlated (or non-independent) due to patient-specific characteristics. The Gamma distribution with log link has been demonstrated to adequately model length of stay outcomes [37,38]. These two statistical approaches were used to directly model the skewness of the data.

In addition, for median number of admissions and bed days we used the non-parametric Wilcoxon test of ranks for comparisons, and in doing so did not make any distributional assumptions.

Logistic regression models were used to determine associations with frequent admissions (3) or very frequent admissions (4+) for survey respondents. Univariate logistic models were used to investigate the association of demographic characteristics, conditions (having CHF, COPD, hypertension, diabetes or coronary artery disease), comorbidity measures and survey variables with frequent or very frequent admissions. We decided apriori to retain the demographic variables, gender, age (65-74/75 or older), and the five ACS conditions in the base multivariate logistic model. The Charlson Comorbidity Index was dichotomised into scores of 0–2 or 3 or more, and each survey variable was added singly to the base model in order to assess the impact on frequent or very frequent admissions. Only survey variables which remained significant in the multivariate model were retained. Models including other hospitalisation factors such as length of hospital stay were also examined, but these variables did not contribute significantly to the analyses. All tests of significance used 95% level (p<0.05).

## Results

### Response

A total of 709 patients met the selection criteria but 101 (14%) died and 96 (14%) moved to aged care facilities between being identified in early 2010 and our first mail out in October 2010. Of the 512 patients invited to participate in the survey 33 invited patients died before the second reminder was sent out. 122 patients (23.8% of those invited) consented to participate in the survey. Of the 122 patients sent a questionnaire, 2 died before completing the questionnaire, six withdrew from the study after consenting, and 105 patients (83.6% of the consenters) completed the questionnaire, including 96 who received a follow up phone call. Results are presented for 102 patients as in three cases the survey was incomplete and hospital admissions data were unable to be linked to survey data and these three cases were excluded from the analysis.

Respondents were encouraged to seek help to complete the questionnaire if necessary, but most respondents reported completing it themselves (72%). The survey completion rate for men (63/273=23%) was higher than for women (39/239=16%) (p=0.060, OR=1.54, 95% CI (0.99-2.40).

### Health and social characteristics of survey respondents

The health and social characteristics of the 102 survey respondents with linked data are summarised in Table 2.

**Table 2:** Demographic, Social and Health Characteristics of Survey Respondents (n=102)

**Demographic Attributes**		n	%
Age group (at survey)	65-79	68	67
	≥80	34	33
Gender	Male	63	62
	Female	39	38
**Socio-economic Status and Social Attributes**		n	%
*Socio-economic Status*			
Pension card	Yes	83	81
Income (n=92)	<$20,000 p.a.	49	53
Private health insurance (n=93)	Yes	22	24
Education	No School Certificate or other qualification	42	41
*Social Attributes*			
Car access	No	32	31
Need regular help	Yes	50	49
Have regular care	Yes	65	64
Household composition	Live Alone	35	34
Duke Social Support Index Items – in the previous week:			
Number of times spent with friends/family you don’t live with	None	25	25
Number of times talked to someone on the phone	None	6	6
Number of times attended meetings, social clubs or groups	None	58	57
Number of people close by that you can depend on	None	8	8
		mean	SD
Social Network score(DSSI)		8.25	1.8
**Health Attributes**			
Overall health rating	Poor	28	28
	Fair	41	40
	Good/Very Good/Excellent	33	32
Falls (in last 12 months)	At least 1	33	32
Quality of life	Poor	16	16
	Fair	32	31
	Good/Very Good/Excellent	54	53
Psychological distress (K10)	Well	48	47
	Mild Disorder	22	22
	Moderate Disorder	24	24
	Severe Disorder	7	7
		n	%
Medication Adherence	Forgetting to take medicines	19	19
	Careless with taking medicines	7	7
	Feel better so stopping medication	3	3
	Feel worse so stopping medication	6	6
	Using a Webster pack	35	34
	Sometimes trouble affording medication	22	22
		mean	SD
Limit daily activities SF36-Physical		42.1	11.9
Medical Condition Self-management (PIH)		1.29	1.4

The mean age of respondents (at survey) was 77.1 years (range 66–95 years). More than four out of five (81%) survey respondents had a pension card and more than one half (53%) had an income of less than $20,000 per year. Forty-one percent had no formal educational qualifications and even though education level did not differ statistically by gender more men (44%) than women (37%) in the survey group had left school before school certificate stage.

Thirty-one percent of respondents reported having no access to a car they could drive, including 29% of those living alone. Of the 50 respondents who said they needed help to care for themselves, 11 (22%) did not have either a close friend or relative who regularly cared for them. This was significantly fewer respondents compared to those who did not need help and did not have either a close friend or relative who regularly cared for them (26 of 52, 50%) (p=0.003).

Overall thirty-four percent of respondents lived alone including 48% of women and 29% of men (p=0.100). Male respondents had slightly worse social network scores (mean=8.0, sd=1.9) than female respondents (mean=8.6, sd=1.6) but the difference was non-significant (p=0.089). The respondents who reported being least socially isolated were women who lived alone. The vast majority of respondents had at least one telephone contact in the previous week, although 6 patients (all male) did not have any. Of the twenty five respondents who had no time in the previous week spent with friends or family they didn’t live with, 19 (76%) also attended no meetings, social clubs or groups with 6 of these respondents living alone. Eight percent of respondents reported having no one outside the home but within one hour of travel, that they felt they could depend on or felt close to. These eight did not live alone.

The majority of survey respondents (68%) rated their health as either poor or fair, in contrast to the 32% who rated their health as good, very good or excellent. These ratings differed significantly by gender (p=0.033), with 36% of men rating their health as poor compared to 16% of women (p=0.026). Slightly more men (56%) than women (39%) had more than two chronic conditions (p=0.090). Almost one third of respondents (32%) had experienced at least one fall in the past year. Most rated their quality of life as good or better (53%) or fair (31%), with only 16% giving a poor rating.

The majority of respondents (53%) had mild, moderate or severe psychological distress (scoring over 15 on the K10 assessment), while 31% of respondents had moderate or severe psychological distress. Sixty percent of respondents in the 65–74 age group, and 46% in the 75 or older groups were in this category of risk but this difference was not statistically significant. We also dichotomised age to 65–79 years and 80+ at time of survey, and found that fewer older (80+ years) respondents had moderate psychological distress than younger respondents (p=0.050). This finding was also reflected in the continuous K10 Psychological distress score with the younger (65–79) group scoring significantly higher (p=0.017) with a mean of 9.6 (SD=7.6) compared to the 80+ group mean of 5.8 (SD=5.6).

Thirty four percent of respondents used a Webster pack (an individually-prepared blister pack of medications). Men with a Webster pack reported forgetting to take their medications more than men without a Webster pack (34.8% vs 16.2%; p=0.100), although the difference was not statistically significant.

Sixty percent of survey respondents had a composite disease self-management score less than 2, indicating that most had reasonably good self-management. No respondent indicated poor self-management (all composite scores were less than 6).

All respondents had a general practitioner (GP). Thirty-eight percent reported having a written plan for looking after their long term health condition. One third reported getting regular reminders to visit their GP.

### Telephone follow-up

Ninety six of the 102 survey respondents received a follow-up telephone call. In response to questions about the determining factor which made them decide to go to hospital for their most recent admission, most respondents (n=67, 70%) described pain and/or breathing difficulties. Eight (8%) respondents reported that anxiety (on its own or alongside pain and/or shortness of breath) was the determining factor that made them decide they needed help. In twelve cases (13%) respondents’ GPs helped them make the decision to go to hospital. Fifty nine respondents (61%) reported they believed nothing could have helped to keep them out of hospital at that time. Seventeen respondents (18%) reported being “coached” to go to hospital when experiencing the specific symptoms that triggered their most recent admission.

Suggestions that some admissions were potentially avoidable arose in a small number of narratives. For example, eleven respondents (12%) described an important relationship between medication and their admission (difficulty accessing medication, taking the wrong medication, finally getting prescribed the right medication, not taking medication when they should have). Four respondents (4%) reported that if they had looked after themselves or ‘followed doctor’s orders’, then they may not have ended up in hospital. Two (2%) reported their admissions were due to over-extending themselves physically. One respondent, in discussing what could have helped them at the time of their most recent admission, reported that they had been instructed regarding breathing exercises on their last hospital visit but had forgotten what the exercises were upon leaving and that this had contributed to their most recent admission.

### Frequent admissions (3) compared to very frequent admissions (4+)

Results of univariate and multivariate logistic regression modelling of frequency of admission are shown in Table 3. The demographic and disease variables were retained in the models regardless of significance. Only significant survey-based variables were retained in the multivariate logistic model. Both the Charlson Comorbidity Index (OR=3.85; 95% CI 1.01-14.7) and the Dukes network score (OR=0.75; 95% CI 0.56-0.99) were significant associations in the multivariate model. Having a Charlson Comorbidity Index of 3 or more nearly quadrupled the odds of having 4 or more admissions, and a reduction of one point on the Dukes network score increased the odds of being a very frequent admitter by 35%. Having chronic heart failure also increased the odds of being admitted very frequently nearly five-fold.

**Table 3:** Characteristics associated with frequent/very frequent admissions: survey respondents (n=102)

		**Univariate logistic regression**				**Multivariate logistic regression**			
**Variable**	**Categories**	**p-value**	**OR**	**95% CI**		**p-value**	**OR**	**95% CI**	
Gender	female/male	0.185	0.56	0.24	1.32	0.917	0.95	0.33	2.69
Age at survey	75+/65-74	0.779	0.89	0.40	2.01	0.825	0.89	0.32	2.46
Charlson comorbidity	3+/0-2	0.010	3.98	1.40	11.29	0.049	3.85	1.01	14.70
CHF	yes/no	0.001	4.14	1.76	9.77	0.003	4.76	1.70	13.31
COPD	yes/no	0.076	2.10	0.93	4.77	0.082	2.69	0.88	8.20
Hypertension	yes/no	0.207	0.58	0.25	1.35	0.098	0.36	0.11	1.21
Coronary artery disease	yes/no	0.605	0.80	0.35	1.84	0.322	1.79	0.57	5.69
Diabetes	yes/no	0.666	1.20	0.53	2.70	0.863	1.10	0.36	3.38
Duke social network	continuous	0.081	0.82	0.65	1.03	0.040	0.74	0.56	0.99
Need help with daily tasks	yes/no	0.017	2.77	1.20	6.40				

Only significant survey-based variables were included in the multivariate logistic model.

### Survey respondents compared to survey non-respondents

We linked the hospital admissions data and North Coast Connecting Care Program data to the survey data for all patients where this link was possible. This enabled us to compare a range of factors for patients in our survey group with those who did not participate in the survey. Tables 4 and 5 compare this linked data for survey respondents (n=102) compared to non-respondents (n=410).

**Table 4:** Characteristics for invited patients (n=512), by response status

		**Survey respondents**		**Survey non- respondents**		**P value**
		**N=102**		**N =410**		
		**n**	**%**	**n**	**%**	
Age group (at survey)						<0.001
	65-79	68	66.7	177	43.2	
	≥80	34	33.3	233	56.8	
Gender						0.056
	Male	63	61.8	210	51.2	
	Female	39	38.2	200	48.8	
Maximum Charlson						0.385
Comorbidity Index	0	28	27.5	88	21.5	
	1-2	55	53.9	230	56.1	
	≥3	19	18.6	92	22.4	
Number of ACS						0.942
conditions (multi-morbidity)	1	18	17.6	67	16.3	
	2	34	33.3	143	34.9	
	3	30	29.4	132	32.2	
	4	17	16.7	58	14.1	
	5	3	2.9	10	2.4	
≥5 medications						0.060
	Yes	13	12.7	86	21.0	
**For Unplanned Admissions in index year**						
Frequent /very frequent						0.067
admissions	3 admissions	65	63.7	220	53.7	
	4+ admissions	37	36.3	190	46.3	
Number of admissions ^1^	Mean(se)	4.1 (0.2)		4.6 (0.1)		0.032
Number of admissions^2^	Median (range)	3 (3–9)		3 (3–11)		0.078
Number of Unplanned Bed Days^3^	Mean (se)	5.2 (0.2)		6.2 (0.3)		0.014
Number of Unplanned Bed Days^2^	Median (range)	4 (1–25)		4 (1–48)		0.040

^1^ Results from Poisson regression model :estimated means, standard errors and p value.

^2^ Results of non-parametric Wilcoxon test of ranks : p values.

^3^ Results of GEE Gamma regression model: stimated means, standard errors and p value.

**Table 5:** Diagnoses for unplanned hospital admissions for invited patients (n=512), by response status

	**Admissions per patient**			**Bed days per admission**		
	**Respondents (n=102)**	**Non-respondents (n=410)**		**Respondents (n=421)**	**Non-respondents (n=1900)**	
	**Mean (se)**	**Mean (SE)**	**P value**^**1**^	**Median [max]**	**Median [max]**	**P value**^**2**^
**Primary Diagnosis**						
Coronary artery disease	0.97 (0.10)	0.84 (0.05)	0.193	3 [18]	3 [35]	0.339
COPD	0.84 (0.09)	0.84 (0.05)	0.929	5 [22]	5 [35]	0.466
Other Cardiovascular	0.29 (0.05)	0.31 (0.03)	0.860	6 [21]	5 [102]	0.650
Chest Pain (non-cardiac)	0.26 (0.05)	0.19 (0.02)	0.154	2 [18]	1 [43]	0.547
CHF	0.23 (0.05)	0.46 (0.03)	0.002	3 [31]	5 [57]	0.047
Diabetes	0.22 (0.05)	0.23 (0.02)	0.943	5 [38]	5 [55]	0.938
Hypertension	0.01 (0.01)	0.04 (0.01)	0.202	1 [1]	2 [7]	0.500
Influenza & Pneumonia	0.19 (0.04)	0.18 (0.02)	0.861	8 [19]	7 [30]	0.856
Other Respiratory	0.08 (0.03)	0.21 (0.02)	0.010	5 [29]	5 [38]	0.879
All other diseases	1.02 (0.10)	1.33 (0.06)	0.018	4 [36]	4 [57]	0.561
**Primary and secondary diagnosis**						
Coronary artery disease	1.63 (0.13)	1.48 (0.06)	0.272	3 [28]	3.5 [53]	0.189
Hypertension	1.42 (0.12)	1.60 (0.06)	0.198	4 [38]	5 [102]	0.095
COPD	1.35 (0.12)	1.34 (0.06)	0.898	5 [31]	6 [57]	0.484
Diabetes	1.19 (0.11)	0.98 (0.05)	0.066	4 [38]	4 [102]	0.914
Congestive Heart failure	0.75 (0.09)	1.03 (0.05)	0.009	5 [31]	6 [57]	0.392
Any targeted ACS chronic admission (primary or secondary)	3.68 (0.19)	3.90 (0.10)	0.293	4 [38]	4.5 [102]	0.040

^1^ Results from Generalised Poisson Linear model with Log link.

^2^ Results from non-parametric Wilcoxon test of ranks.

Survey respondents were younger than non-respondents. The mean age at survey was significantly older (p<0.001) for non-respondents (81.2 years, range 66–100) by on average 4.1 years, than the respondents (77.1, range 66–95), and a larger proportion of non-respondents were 80 years or more (56.8% versus 33.3%).

Of the 512 patients invited 250 (48.8%) had 3 or more of the five targeted chronic conditions and this did not vary by respondent group. Fewer respondents reported five or more medications (12.7%) compared to non-respondents (21%) but this was not statistically significant. All of the 512 patients invited to participate in the study were registered with a GP.

The proportion of respondents and non-respondents with four or more unplanned admissions did not vary. The estimated mean number of unplanned admissions, and bed days (length of stay) were significantly higher for non-respondents than respondents, using the Poisson (p=0.032) and GEE gamma (p=0.014) models to handle the inherent skewness of these variables. The median number of unplanned admissions (3) did not differ significantly between the non-respondent group and survey group (p=0.078), however the median length of stay (number of bed days) differed significantly between groups (p=0.040) using the non-parametric Wilcoxon test.

Primary and secondary diagnoses for respondents and non-respondents are presented in Table 5. For both groups, the most common primary diagnoses were coronary artery disease and COPD. For primary and secondary diagnoses combined, these conditions were again the most frequent, together with hypertension. For patients with a primary diagnosis of CHF, the survey non-respondent group differed significantly, with higher mean number of admissions (p=0.002) and higher median number of bed days (p=0.047) from the survey respondent group. Also, for patients with CHF as a combined primary and secondary diagnosis the mean number of admissions was higher for non-respondents than for respondents (p=0.009). The survey non-respondents had significantly higher median bed days (p=0.040) for any ACS condition when compared to the survey group.

One quarter (24.9%) of all unplanned emergency admissions were for one day only. In the respondent group, 111 admissions (26.4%) were for one day (mean one day admission per patient of 1.09) and in the non-respondent group 473 admissions (24.9%) were for one day. The proportion of unplanned admissions which were for one day was not statistically different between the two groups (p=0.922).

## Discussion

Our survey results describe an elderly rural group of patients with multiple hospital admissions for chronic disease. This group had high rates of comorbidity and a large proportion entered aged care or died within the study period. The group were predominantly of lower socio-economic status, and social isolation and psychological distress were common. Factors significantly associated with more frequent admission (4+ admissions compared to 3 admissions) were a diagnosis of CHF, a lower social network score i.e. respondents with smaller social networks and with less social contact, and a higher Charlson Comorbidity Index score. All patients in the invited group had a GP and our results suggest that at least from the patients’ perspectives their hospital admissions were appropriate and unavoidable on the day. Fewer than half our respondents had a written GP care plan.

Significantly fewer of our survey respondents rated their general health as good or better compared to the North Coast population aged 75 and over [39] (32% vs 72% p<0.001). Generally, respondents were of low socio-economic status which is consistent with the literature [16,40]. Most respondents were on a pension (81%) and this rate is significantly higher (p=0.001) than for the 65+ population in the North Coast Area Health Service area in 2009 (69%) [39]. Nearly one quarter (22%) of respondents reported difficulty affording their medication with important implications for the treatment of their condition and for avoiding hospital admission.

We anticipated that medication would be an important factor in frequent hospitalisation for this group based on the literature [41-43] and previous work by our group [26]. One third (34%) of the invited group (n=512) were using five or more medications (defined as ‘polypharmacy’ by Linjakumpu et al. [44]), the complexity of which may lead to interactions and/or medicine mismanagement. Nearly a quarter of men reported ever having forgotten to take their medication. Interestingly, men with a Webster pack reported forgetting more than men without a Webster pack (34.8% vs 16.2%). This may be because those who were having difficulty remembering their medications were more likely to be offered these packs. Interventions such as the Home Medicines Review (HMR), conducted in the community in Australia, have been shown to be effective at reducing potentially inappropriate medicines in older age groups [45], which might lead to hospitalisation. Greater access to HMR in rural areas via GPs (particularly given this patient group appear to have GPs) might make a contribution to improving individual medication management, although access to pharmacists in rural areas can be problematic, and increased access to HMRs does not address the issue of affordable medication. We had no evidence of the utilisation rates of HMR in our respondents.

Social isolation was an important factor for many of our respondents. We were not able to explore social isolation in non-respondents as it is not routinely collected in hospital admission data. One third (34%) of respondents lived alone (more commonly women). This is significantly higher (p=0.018) than in the general population where 25% of older people (60+) in NSW live alone [46]. The social network scores amongst our respondents indicated greater social isolation than in a recent study of Australian women 70–85 years who had experienced a major illness in the last 12 months, or who had a probable psychiatric condition [47]. One quarter (25%) of respondents spent no time with friends/family they didn’t live with in the previous week. Of the 35 respondents who lived alone, six of these (all men), had no social contact with friends/family nor attended meetings or clubs (their median Dukes network score was 6) in the previous week. Consistent with other studies [47], the least socially isolated respondents were women living alone. Previous work has shown social isolation associated with poor health, depression and loss of confidence [48]. A recent qualitative study reported that fifty six out of one hundred elderly people interviewed who presented to an Australian city public hospital emergency department reported feeling socially disconnected [49]. Our own qualitative study with a broad range of community-based health and support service providers suggested that social isolation was a key factor contributing to frequent and/or avoidable hospital admissions amongst older patients with chronic disease [26]. This is supported by the finding that among survey respondents, greater social isolation was associated with having more frequent admissions (4+ admissions). The possible complex mechanisms whereby social isolation might contribute to admission in older rural patients with chronic disease, for example social isolation leading to depression leading to lack of motivation for self-management, are discussed in our paper on this topic [50].

A number of related social factors were identified which may be contributing to the high use of hospital services. Nearly one third of respondents reported having no car they could drive and of those living alone, 29% had no car access. This has implications not only for exacerbating their social isolation but also for accessing services in rural Australia where public transport is limited or non-existent, if they have no friends or family to take them. One in five (22%) of respondents who reported needing regular help with daily tasks e.g. personal care, did not have either a close friend or relative who regularly cared for them. While these patients may have obtained carer support from community or social services, a degree of unmet need is likely to be present.

There are a number of community based services which might partially ameliorate the impact of social isolation including valuable community transport initiatives, however, many of these services have been reduced in recent years e.g. services which used to offer accompanied shopping trips now only offer unaccompanied shopping [51]. These services often have long waiting lists and there are often considerable additional barriers to accessing services, sometimes specific to rural locations [51,52]. Increasing this provision and access to it, may present an opportunity to impact on social isolation which may in turn affect hospital admission amongst this patient group.

Over half the respondents (53%) had mild, moderate or severe psychological distress (scoring over 15 on the K10 assessment). With 60% of those aged 65–74 years and 46% of those 75 or older experiencing psychological distress. This is significantly higher than for the corresponding age groups in the general Australian population (25%, p<0.001; and 29%, p<0.001, respectively) [53]. This finding points to possibly undiagnosed psychological distress amongst this group. Psychological distress may contribute to hospitalisation through poor chronic disease management due to low incentive and energy to self-manage [54,55]. This suggests that improved GP attention to the mental health status of patients with chronic disease and a history of frequent admission might be beneficial in both diagnosing and treating mental health disorders, as well as activating self-management [56]. However, the majority of survey respondents rated their self-management as reasonably good.

While all the study population were registered with a GP, we had no information on the nature or frequency of contact with their GP. Only one in three (34%) survey respondents recalled receiving regular reminders to visit the GP, and only 38% reported having a written care plan. Our study population is composed of the type of patient for whom care plans are thought to be particularly beneficial. This suggests a failure of delivery of best practice in primary care amongst this group which could possibly be improved and warrants further exploration.

In our study population COPD and coronary artery disease were the most common primary diagnoses. A higher Charlson Comorbidity Index score was associated with more frequent admission, as was congestive heart failure. Given that congestive heart failure is characterised by progressive deterioration, frequent hospitalisation and requires complex self-management, this is not surprising [41]. An ethnographic study of self-management by people with diabetes has described the difficult balancing act required, and that capacity to self-manage was limited for some people by their co-morbidity, psychological factors and social capital, all issues among our population [57]. While the specifics of self-management for diabetes are different to those for heart failure, they are similarly complex, require close self-monitoring, careful attention to diet and medications, and impose important social restrictions. In North Coast NSW there is a well-established and regarded Heart Failure Program, and evidence exists demonstrating that programs for CHF can reduce hospitalisation in the short term amongst specific groups [58]. However, difficulties with these kinds of programs relate to access, particularly in rural areas, and ‘maintenance’, a crucial factor with all chronic disease but particularly with CHF. Programs run for a finite timeframe of a few weeks of contact per patient, whereas many patients require longer-term regular on-going support to sustain their motivation and capacity for modifying health behaviours (for example monitoring fluid intake), and/or to account for the fact that these patients often have poor short-term memory and some dementia [51].

The high mortality amongst this group represents a significant study challenge. In our study nearly one in five (n= 136, 19%) patients of the initially identified 709 were known to have died within 2 and one half years (between July 2008 and January 2011). Since the commencement of the Connecting Care Program (and the identification of our study population) the eligibility criteria for the Program has been amended to identify patients at the hospital when they are admitted rather than retrospectively (as was the case in this cohort), to include patients of all ages and to lower the threshold of multiple admissions from the 3 plus used initially [59]. This will likely target patients less ill than those in the study population we report in this paper, and may extend the scope for improving disease management and thus reduce future potentially avoidable admissions.

### Avoidability

Given the level of morbidity of respondents, and that some patients had been “coached” to present to the Emergency Department under particular circumstances, it is unsurprising that the majority reported admissions as appropriate on the day, that is, from the patient’s perspective the admission could not have been avoided on that day. This highlights the difficulties of using ACS admissions as a proxy measure for “avoidability”.

Whilst our telephone follow-up focused on appropriateness of admission on the day, we also obtained a small number of unprompted narratives around potential longer-term avoidability of admission. These included medication issues and anxiety, and health behaviours such as not following ‘doctors orders’ or physical over-exertion, although no strong themes emerged from this limited information. Future work should include an exploration of avoidability in the weeks and months leading up to a specific admission, and investigate the decision-making process which leads patients to call for an ambulance [2], to ensure the patient and clinician perspective of potential avoidability of hospitalisation is adequately captured.

### Response rate

We anticipated that the study population would be difficult to engage in a written survey due to their age and morbidity. While only one quarter (24%) of those invited consented to participate in the study, a high proportion of consenters completed the survey questionnaire (105/122 86%) with only nine patients not responding. Two consenting patients died before completing the questionnaire and six patients withdrew from the study after consenting mainly due to deteriorating health, reinforcing our experience of this group as vulnerable and sick.

A number of mechanisms were employed to maximize the survey response rate including personalising letters, providing a return envelope and follow up telephone contact [60]. We also used coloured paper and photographs of two of the research team on the front cover of the questionnaire and anecdotal evidence from contact with patients suggested that these two elements improved the response.

The higher proportion of men who completed the survey is unexpected from other general household survey research using postal questionnaires [61] but is similar to findings from previous surveys amongst older patients with chronic disease [62].

### Limitations

Our respondents were younger, and were less sick (they had significantly fewer unplanned admissions and shorter stays in hospital) than non-respondents. Non-response has previously been associated with morbidity, age, and with living alone [61,62]. Although the generalizability of our survey results to the larger study population is limited by the low response rate and restricted sample size, it is likely that our results offer a conservative picture of the study population, and that the potentially unmet needs in the respondent group apply to the non-respondent group. We acknowledge that the invited group were selected from the public hospital system, thus presenting a potentially biased picture of older people with chronic conditions and a history of frequent admission. However, our understanding is that private hospitals in the region rarely deal with unplanned hospital admission which was the focus of our study.

Cross-sectional surveys are limited by capturing only a moment in time, and our understanding of causal associations is limited by reverse causality, a feature of this type of study design. Surveys restrict the information respondents provide, by what they do not include, and by nuance of interpretation by respondents. Some of these limitations were redressed by the telephone follow up that we conducted. As this was a preliminary study, our intention in future work is to conduct more robust and in-depth qualitative work with this patient group to ensure the patient perspective is fully captured.

We assumed that the level of cognitive impairment in our respondent group would be less than in our non-respondent group, but had no means of assessing this. If this was the case, the implications of our work, particularly for self-management (including medications management), would be magnified amongst the non-respondent group. Finally, a major limitation of our study was that the small sample size of the survey respondents limited our ability to detect any but large differences between sub-groups.

## Conclusion

This study provides a detailed description of older patients dealing with multiple chronic conditions and a history of frequent admission in rural Australia. While the disease severity amongst this group likely limits opportunities to reduce hospital admissions through improved community-based care, some situations were identified in which admission might have been avoided through greater medication adherence and modified health behaviours. This study also identified a number of areas of potentially unmet need amongst this highly vulnerable population including difficulty affording medication, difficulty in accessing services in rural areas, poor medication management, social isolation and mental health issues. Greater GP input into care planning, education and behaviour modification may help. Addressing these areas may have the potential to impact on frequent hospital admission amongst this patient group.

## Competing interests

The authors declare that they have no competing interests.

## Authors’ contributions

JL, KH, MP, DE, LB and GM designed the study. JL, KH, MP, GM, LB and DE designed the questionnaire and managed its completion and recording. JL and KH conducted, managed and analysed follow up phone calls. TD and MR managed the survey data and linked the data, and MR conducted the statistical analysis. JL, MR, MP, DE and MP produced the first drafts of the manuscript and all authors contributed comments and acknowledged authorship.

## Authors’ information

This project was initiated by the concerns of staff in our local general practice network and public health service. Two of our investigators on the project have joint appointments between the Local Health District and the University Centre for Rural Health.

## Pre-publication history

The pre-publication history for this paper can be accessed here:

http://www.biomedcentral.com/1472-6963/12/373/prepub

## Supplementary Material

Additional file 1 **Appendix 1.** Additional material on survey questionnaire measures. Click here for file
