# Supplementary material for: Frequent hospital admission of older people with chronic disease: a cross-sectional survey with telephone follow-up and data linkage
Source: BMC Health Serv Res. 2012 Oct 30;12:373. doi: 10.1186/1472-6963-12-373 (PMC3504579; doi:10.1186/1472-6963-12-373)
Supplement: Additional file 1 — Appendix 1. Additional material on survey questionnaire measures. [file 1472-6963-12-373-S1.doc]

# Appendix 1 (MS 2056106097759091) – Additional material on survey questionnaire measures

Functional health and well-being was based on 9 of the 10 items from the SF-36 (version 2). The omitted item related to ‘vigorous activity’ which is not generally applicable to our group of patients. Scores were adjusted for the reduced number of items. Overall health rating and quality of life questions included “In general how would you rate your overall health?” and “In general how would you rate your Quality of Life?”.

The abbreviated Duke Social Support Index (DSSI) , was used to obtain information on social network and asked the number of occurrences in the last week where: time was spent with friends, telephone conversations took place, and meetings/social clubs/groups were attended as well as the number of nearby persons one could depend on. The scoring system used in our study followed that described in Pachana *et al* 2008 . Psychological Distress was measured using Kessler 10 which has been widely used in the Australian context.

Patients reported on their pattern of medication adherence. Items assessed included: forgetting to take medication; being careless at times about taking medicine; feeling better so sometimes stop taking medicine; sometimes feel worse so stop taking medicine. Other medication adherence questions included Webster pack use.

The Partners in Health Scale (PIH) self-management assessment of chronic disease was used to rate self-management of condition. It includes items on knowledge of the treatment; ability to arrange appointments; attendance at appointments; and understanding of what to do if symptoms worsen. It is scored from 0 to 8 (where 0 is very good and 8 is very poor). This scale has been used in chronic disease programs in Australia for older people.

#### Questionnaire Measures

**Limit On Activities** (Q7) uses 9 of the 10 items (on a 3 point scale) from the well validated and reliable Physical Function Subscale from the SF-36 v2 Health Survey . The omitted item related to ‘vigorous activity’ which is not generally applicable to our group of patients. Higher scores indicate better physical functioning. Scores were adjusted for the reduced number of items. We transformed the original percentage scores with respect to age-specific Australian norms to T scores which have a mean of 50 and SD of 10. T scores enable direct comparisons irrespective of age.

**Health Rating** (Q8) uses a composite variable of ‘self-report health rating’ calculated from: Overall health, Quality of life, Eyesight, Memory and Teeth/gums on a five point scale (4=Excellent, 3=Very Good, 2=Good, 1=Fair and 0=Poor). A mean of these five items was used to obtain a composite score for self-reported health rating, with higher scores indicating a better health rating. The single items of overall health, and quality of life, are also reported as a 3 level item with categories Excellent, Very good and good combined into ‘Good/Excellent’, and Fair/Poor.

**Social Network**(Q11, Q12): This is the four item social interaction or network subscale from the validated abbreviated Duke Social Support Index (DSSI) , and include number of occurrences in the last week where time was spent with friends, with telephone conversations and attending meetings as well as the number of nearby people one could depend on. The scoring system used in our study followed that described in the supplementary material from Pachana et al 2008 . After scoring the items were summed to obtain a network score ranging from 4 to 12 where higher scores indicate a larger social network or less social isolation.

**Psychological Distress (Kessler 10)** (Q13): This instrument is a well validated and reliable measure of psychological distress and has been widely used in the Australian context for state and national based population surveys. Each of the 10 items is scored on a 5 point scale (1-5) and the composite summed total score could range from 10 to 50. Higher scores indicate higher levels of psychological distress. Cut-offs from the Australian Bureau of Statistics of Well (10-15), Mild (16-21), Moderate (22-29) and Severe (30 or greater) psychological distress were used.

**Medication Adherence (**Q16): reports binary scores (Yes/No) from: ever forget to take medication, being careless at times about taking medicine, feel better so sometimes stop taking medicine, sometimes feel worse so stop taking medicine . This measure has been developed for use with patients with hypertension (one of our targeted conditions). Other indications of medication adherence included Yes/No questions on using a Webster pack (a blister pack setting out medicines for each particular time of the day, for each day of the week constructed for an individual patient) , and trouble affording medication.

**Self- management of Condition** (Q17): five items as a subset of the 12 items from the Partners in Health Scale (PIH) were used to assess self-management of chronic disease . This scale has been used in chronic disease programs in Australia for older people. A composite variable of ‘condition self-management’ was calculated as the mean of the 5 items, each scored from 0 to 8, (where a 0 score indicated ‘very good‘, while 8 indicates ‘very poor’). These items questioned knowledge of the condition, knowledge of the treatment of condition, ability to arrange appointments, attendance at appointments, and understanding of what to do if symptoms worsen. The PIH can be reported as a mean of all item scores .

**Socio-economic status:** Responses to survey questions on education Q3, income Q24, holding a pension card Q25 and having private health insurance Q25 constitute the variable socio-economic status (SES), based on Australian Bureau of Statistics factors relating to older people . For reporting purposes these variables were dichotomised to: Education (No qualifications/School certificate and higher); Income (Less than $20,000/$20,000 or more); Pension card (Yes/No) and Private health insurance (None/Yes to any private health insurance questions ).

Questions on access to a car you can drive, regularly needing help with daily tasks, having someone who regularly cares for you with Yes/No responses are designed to identify access and care difficulties. The incidence of falls can have a possible impact on unplanned hospital admissions so was included in the questionnaire as ‘during the last 12 months, how many times have you fallen to the floor or ground’. These questions are used in the 45 and Up Study .
